# Swimming and Asthma: Differences between Women and Men

**DOI:** 10.1155/2013/520913

**Published:** 2013-02-20

**Authors:** Marja Kristiina Päivinen, Kari Lasse Keskinen, Heikki Olavi Tikkanen

**Affiliations:** ^1^Unit for Sport and Exercise Medicine, Institute of Clinical Medicine, University of Helsinki, Helsinki, Finland; ^2^Foundation for Sports and Exercise Medicine, Paasikivenkatu 4, 00250 Helsinki, Finland; ^3^Finnish Society of Sport Sciences Olympic Stadium, Paavo Nurmentie 1B, 00250 Helsinki, Finland

## Abstract

*Background and Aim*. Asthma is common in endurance athletes including swimmers. Our aim was to study gender differences in asthma, allergy, and asthmatic symptoms in swimmers and investigate the effects of varying intensities of physical exercise on competitive swimmers with asthma. *Methods*. Three hundred highly trained swimmers (156 females and 144 males) were studied by a questionnaire. Their mean (±SD) ages were 17 ± 3 and 19 ± 3 years, and they had training history of 7 ± 2 and 7 ± 3 years in females and males, respectively. Gender differences in asthma, allergy, and respiratory symptoms were examined. Special attention was focused on asthmatic swimmers, their allergies and respiratory symptoms during swimming at different intensities. *Results*. The prevalence of physician-diagnosed asthma was 19% for females and males. No gender differences in asthma or respiratory symptoms were found. Males reported allergies significantly more often than females (*P* = 0.007). Gender difference was found in respiratory symptoms among swimmers with physician-diagnosed asthma because females reported symptoms significantly more often (*P* = 0.017) than males. Asthmatic females also reported symptoms significantly more often at moderate intensity swimming (*P* = 0.003) than males especially for coughing. *Discussion*. Gender difference in prevalence of asthma was not found in swimmers. However, allergy was reported significantly more by male swimmers. Male swimmers with asthma reported significantly more cases having family history of asthma, which may be a sign of selection of asthma-friendly sport. Moderate intensity swimming seemed to induce significantly more symptoms especially coughing in asthmatic females.

## 1. Introduction

The prevalence of asthma among Finnish competitive swimmers is higher than that in general population which is typical in endurance sports [1, 2]. Gender differences in prevalence of asthma have been found in general population. Asthma and especially the nonallergic asthma were more prevalent in women than men [3, 4]. In Finnish general population asthma was mostly associated with family history of asthma, allergy, smoking, and obesity [4]. General population is different than competitive swimmers population, because weight gaining, aging, and smoking-related factors are missing as competitive swimmers are mainly fairly young, with normal BMI and nonsmoking. 

Intensity, duration, and type of exercise are known to have an effect on the severity of the respiratory symptoms [5]. For example, at the same exercise intensity level, asthmatic symptoms are fewer and milder in swimming than in running or cycling [6, 7]. That may affect the selection of sport in persons who have sensitivity in lung function. In all endurance sports like swimming as well, the demands for lung function are high due to prolonged workouts in high intensities. 

In asthmatics physical exercise in the intensity level of 90% of maximum heart rate for 6–8 minutes typically causes the asthmatic symptoms [8]. In a previous study by Päivinen et al. (2010) [9], reported respiratory symptoms in five different swimming intensities were studied. To our knowledge the gender differences in asthmatic symptoms in different intensities in swimmers with physician-diagnosed asthma are not studied before.

The aim of the study was to examine gender differences among competitive swimmers and especially swimmers with asthma. The focus was to concentrate on comparing allergies, family history of asthma and allergies, and the respiratory symptoms in different intensities in physical exercise between female and male swimmers. As swimming has been suggested to be a suitable physical exercise therapy for persons with asthma, this study examined swimming special features related to asthma and allergy to obtain further knowledge of the differences between female and male swimmers.

## 2. Methods

Three hundred Finnish competitive swimmers, 156 females and 144 males, were studied by a questionnaire. Studied swimmers were selected for the study according to the sufficient training history and performance level of qualification for national championships. Swimmers were at mean SD age of (mean SD) 18 ± 3 years, females 17 ± 3 and males 19 ± 3 years. Swimming training history was mean 7 ± 2 years in females and 7 ± 3 years in males. Weekly training hours were on the average 16 ± 4 hours in females and 16 ± 3 hours in males. 

Gender differences of prevalence of physician-diagnosed asthma, allergy, and respiratory symptoms were studied first. Then, the swimmers reporting physician-diagnosed asthma were taken into further examination. The differences were studied with the chi-square test and considered significant when *P* value was <0.05. Swimmers with physician-diagnosed asthma were subjected to further detailed analysis. Wilcoxon sum rank test was used to compare the ages of the onset of asthma diagnosis in asthmatic swimmers.

The questionnaire consisted of questions concerning basic information, training history, asthma, allergy, family history of asthma and allergy, and respiratory symptoms (shortness of breath, wheezing, coughing, and mucous production) during swimming. Five (I–V) different swimming intensities as presented, for example, by Keskinen 1993 [10] were recorded. Intensity zone I represented easy endurance swimming, zone II was moderate endurance exercise between aerobic and anaerobic thresholds, zone III was a swimming pace between the anaerobic threshold and the minimal velocity to achieve maximal oxygen uptake, zone IV velocity was higher than zone III, with a competition-specific race pace maximizing muscle lactic acid production, and zone V was all-out sprinting [10]. The questionnaire has been applied earlier for swimmers [9]. 

In asthmatic swimmers, physician-diagnosed allergy, family history of asthma and allergy, and reported asthmatic symptoms were studied in different training intensities. The analysis describing symptoms in five different training intensities was previously used by Leynaert et al. 2012 [3]. Asthmatic symptoms wheezing, coughing, shortness of breath, and mucous production during swimming at five (I–V) different exercise intensities [10]. 

The ethics committee of the Hospital District of Helsinki and Uusimaa approved the study protocol.

## 3. Results

**Figure 1:**
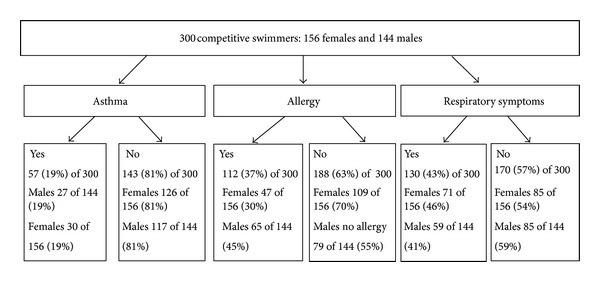
The prevalence of physician-diagnosed asthma, physician-diagnosed allergy, and reported respiratory symptoms in 300 competitive swimmers.

Physician-diagnosed asthma prevalence was 19% in both females and males. No gender differences in prevalence of physician-diagnosed asthma and reported respiratory symptoms were found. Male swimmers reported significantly more physician-diagnosed allergy (*P* = 0.007) than females. No gender differences were found when reporting respiratory symptoms in swimming in the whole 300 swimmers population (Figure 1). 

However among swimmers with asthma, gender differences were found in the age of onset of asthma, when reporting respiratory symptoms, and in the family history of asthma (Table 1). Females reported significantly more symptoms especially at moderate intensity swimming (Table 3). No gender differences were found in allergies in swimmers with physician-diagnosed asthma (Table 2). 

**Figure 2:**
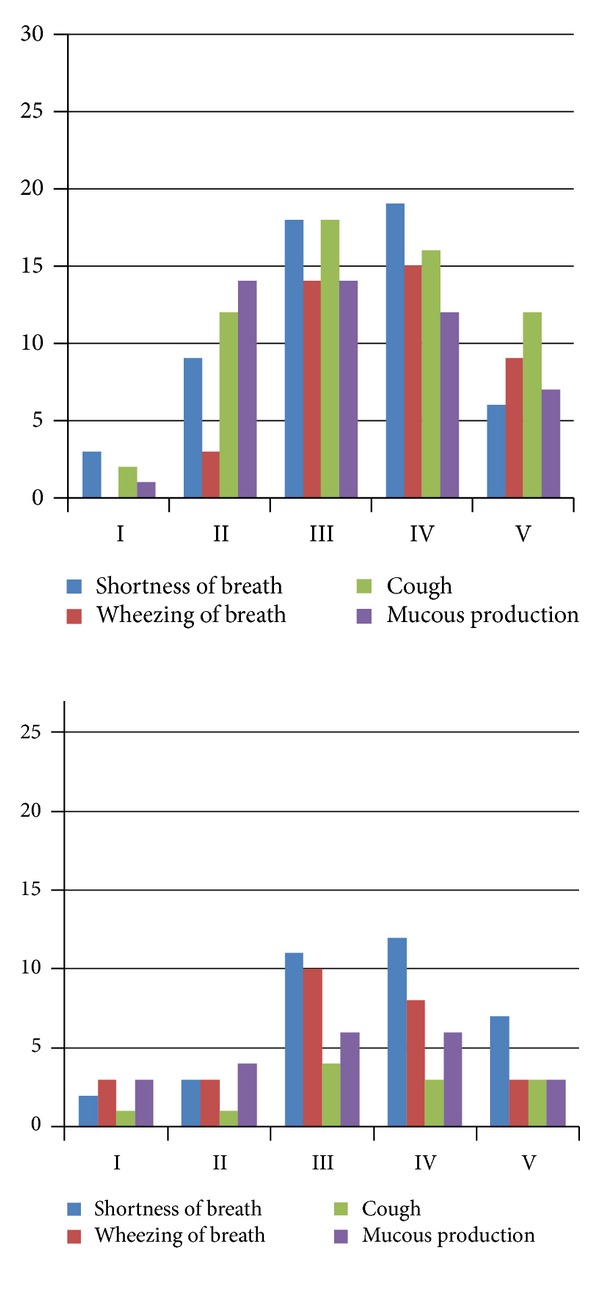
Reported respiratory symptoms at different swimming intensities (I: easy, II: moderate, III: hard, IV: very hard, and V: maximum sprint) in 30 asthmatic female swimmers on the left panel and in 27 asthmatic male swimmers.

In swimmers with asthma, gender difference was observed as females reported more symptoms at the intensity II: moderate endurance training (Figure 2). Female swimmers with asthma reported significantly more coughing and mucous production at moderate swimming intensity. In addition female swimmers with asthma reported significantly more coughing in every intensity level than male swimmers with asthma (Table 4).

## 4. Discussion

Among the studied 300 swimmers, no gender difference in prevalence of asthma was found, unlike in the previous studies in Finnish general population [4]. This finding may be explained by selection of swimming in many ways. Athletes with asthma may choose swimming because it is a low asthmogenic sport [6, 7], and in spite of asthma many athletes may achieve international top level [11]. The studied competitive swimmers had normal weight and were nonsmoking young adults, so the reasons mostly related to gender differences in general population in females were excluded: obesity and hormonal changes in the age of 40–60 [12]. 

Significant gender differences were found in physician-diagnosed allergy. Male swimmers reported more allergies than females. That may be linked to the fact that swimming is a low-symptom-causing sport and therefore especially chosen by males with allergy. In swimmers with asthma no gender differences in reported physician-diagnosed allergy were found. That was inconsistent with previous studies on general population where nonallergic asthma was found more in females [3].

Among swimmers with asthma gender difference was significant in the age of the onset of asthma (Table 1) as male swimmers had asthma in younger age than females. Results show that male swimmers mainly had asthma before swimming training had started and females later during swimming career, almost in puberty. Male swimmers reported significantly more often family history of asthma than asthmatic females. Therefore, fewer cases having family history of asthma and later onset of asthma may be related to higher amount of reported respiratory symptoms during swimming in asthmatic female swimmers.

In previous studies [2, 9] family history of asthma and allergies have been linked to asthma in athletes. Also in this study only two of 57 swimmers with asthma (0.07% of 300 studied swimmers) reported asthma without any allergy or family history of asthma. 

Gender differences in swimmers with physician-diagnosed asthma were found in reported respiratory symptoms during swimming. Interestingly, coughing, which in previous studies has been reported to be the most common respiratory symptom in athletes [5, 13], was frequently reported in asthmatic female swimmers but not in males. In a study by Heir (1994) [14] all but coughing was more frequent in athletes with asthma, which is consistent with the result in this study on asthmatic male swimmers [14]. The results showed, surprisingly, that female swimmers with asthma reported significantly more often symptoms in moderate intensity than males (Figure 2 and Table 4). This observation may have been linked to the asthma background as the females had their asthma diagnosis mainly during swimming career. It may also be influenced by smaller lungs related to body size in females than males. This may also cause gender difference in the ventilation dynamics during swimming and the efficiency in body-size-related work load in physical exercise on land. With different ventilation dynamics in females and males, moderate intensity swimming may be different. 

## 5. Conclusions

According to asthma allergy and respiratory symptoms gender differences in competitive swimmers vary from general population. In swimmers with physician-diagnosed asthma gender differences were seen in reported asthmatic symptoms especially in moderate intensity and coughing. 

## Figures and Tables

**Table 1:** Gender differences in 57 swimmers with physician-diagnosed asthma out of 300 elite competitive swimmers.

Physician-diagnosed asthma	Women 30	Men 27	*P* value
Age (mean SD), years	17 ± 3	19 ± 3	<0.001*
Age of the asthma diagnosis (mean SD), years	13 ± 3	9 ± 6	<0.001*
Allergy	20/30	21/27	0.462
Reported respiratory symptoms	29/30	20/27	0.014*
Family history of asthma	5/30	14/27	0.005*
Family history of allergy	16/30	17/27	0.462

*Significant difference.

**Table 2:** Gender differences in types of allergy and allergic reaction in the asthmatic swimmers.

Physician-diagnosed asthma	Women 30	Men 27	*P* value
Animal allergy	19/30	16/27	0.696
Pollen allergy	13/30	17/27	0.138
Home dust allergy	6/30	11/27	0.87
Food allergy	8/30	7/27	0.949
Allergic reaction in eyes	12/30	10/27	0.819
Allergic reaction as runny nose	17/30	12/27	0.357
Allergic reaction on skin	4/30	6/27	0.378
Other types of allergic reactions	7/30	7/27	0.820

*Significant difference.

**Table 3:** Gender differences of reported respiratory symptoms at different training intensities in asthmatic swimmers.

Reported symptoms at swimming intensity	Women with asthma	Men with asthma	*P* value
Easy: I	8/30	4/27	0.273
Moderate: II	17/30	5/27	0.003*
Hard: III	13/30	13/27	0.716
Very hard: IV	16/30	12/27	0.503
Maximum sprint	14/30	7/27	0.105

*Significant difference.

**Table 4:** Gender differences in reported respiratory symptoms: shortness of breath, wheezing, coughing, and mucous production at different swimming intensities in 30 asthmatic female swimmers (F) and 27 asthmatic male swimmers (M).

	Shortness of breath			Wheezing			Coughing			Mucous production		
	F	M	*P* value	F	M	*P* value	F	M	*P* value	F	M	*P* value
Easy	3/30	2/27	0.730	0/30	3/27	0.061	2/30	1/27	0.913	1/30	3/27	0.251
Moderate	9/30	3/27	0.081	3/30	3/27	0.891	12/30	1/27	0.001*	14/30	4/27	0.010*
Hard	18/30	11/27	0.146	14/30	10/27	0.462	18/30	4/27	<0.001*	14/30	6/27	0.054
Very hard	18/30	11/27	0.146	15/30	8/27	0.118	18/30	4/27	0.001*	12/30	6/27	0.149
Max sprint	6/30	7/27	0.594	9/30	3/27	0.081	12/30	3/27	0.013*	1/30	3/27	0.251

F: females, M: males.

*Significant difference.
